# Novel phosphate deficiency-responsive long non-coding RNAs in the legume model plant *Medicago truncatula*

**DOI:** 10.1093/jxb/erx384

**Published:** 2017-11-20

**Authors:** Tianzuo Wang, Mingui Zhao, Xiuxiu Zhang, Min Liu, Chenge Yang, Yuhui Chen, Rujin Chen, Jiangqi Wen, Kirankumar S Mysore, Wen-Hao Zhang

**Affiliations:** 1 State Key Laboratory of Vegetation and Environmental Change, Institute of Botany, the Chinese Academy of Sciences, P. R. China; 2 Research Network of Global Change Biology, Beijing Institutes of Life Science, the Chinese Academy of Sciences, P. R. China; 3 University of Chinese Academy of Sciences, the Chinese Academy of Sciences, Beijing, P. R. China; 4 Noble Research Institute, USA; 5 Inner Mongolia Research Center for Prataculture, the Chinese Academy of Sciences, P. R. China

**Keywords:** High-throughput sequencing, long non-coding RNAs (lncRNAs), phosphate deficiency, *Medicago truncatula*, phosphate acquisition, legume plants

## Abstract

Emerging evidence indicates that long non-coding RNAs (lncRNAs) play important roles in the regulation of many biological processes. Inhibition of plant growth due to deficiency in soil inorganic phosphate (Pi) occurs widely across natural and agricultural ecosystems; however, we know little about the function of plant lncRNAs in response to Pi deficiency. To address this issue, we first identified 10 785 lncRNAs in the legume model species *Medicago truncatula* by sequencing eight strand-specific libraries. Out of these lncRNAs, 358 and 224 were responsive to Pi deficiency in the leaves and roots, respectively. We further predicted and classified the putative targets of those lncRNAs and the results revealed that they may be involved in the processes of signal transduction, energy synthesis, detoxification, and Pi transport. Finally, we functionally characterized three Phosphate Deficiency-Induced LncRNAs (*PDIL*s) using their corresponding *Tnt1* mutants. The results showed that *PDIL1* suppressed degradation of *MtPHO2,* which encodes a ubiquitin-conjugating E2 enzyme regulated by miR399, while *PDIL2* and *PDIL3* directly regulated Pi transport at the transcriptional level. These findings demonstrate that *PDIL*s can regulate Pi-deficiency signaling and Pi transport, highlighting the involvement of lncRNAs in the regulation of responses of plants to Pi deficiency.

## Teaser

Phosphate deficiency-responsive lncRNAs in *Medicago truncatula* are involved in the regulation of Pi deficiency signaling network and Pi transport.

## Introduction

Improvements in high-throughput sequencing technology have revealed that over 90% of the genome can generate a large number of non-coding RNAs (ncRNAs) (Chekanova *et al.*, 2007; Kapranov *et al.*, 2007). These ncRNAs are categorized into small RNAs and long non-coding RNAs (lncRNAs) according to their length (Brosnan and Voinnet, 2009; Kim *et al.*, 2011). LncRNAs have a length of more than 200 nucleotides and relatively less protein-coding capacity, and constitute the biggest class of ncRNAs (Rinn and Chang, 2012; Chekanova, 2015). The majority of lncRNAs can be transcribed by RNA Polymerase II, and they are generally expressed in a tissue-specific manner (Wilusz *et al.*, 2009). According to their locations relative to protein-coding genes in the genome, lncRNAs can be further grouped into sense, antisense, bidirectional, intronic, and intergenic lncRNAs (Ponting *et al.*, 2009).

The function of lncRNAs has long been ignored, and they have often been regarded as transcriptional ‘noise’ due to their low expression and low protein-coding potential. However, recent studies have provided convincing evidence to support regulatory roles of lncRNAs in numerous biological processes in plants (Chekanova, 2015; Liu *et al.*, 2015). Transcriptional regulation of lncRNAs by regulating their targets is the most common way to modulate biological processes, and the lncRNAs can work either in close proximity (*cis*-acting) or at a distance (*trans*-acting) by sequence complementarity with RNA and DNA (Kornienko *et al.*, 2013). The regulatory mechanisms of lncRNAs include transcriptional interference, chromatin remodeling, promoter inactivation, transcription factor activation, epigenetic silencing, as well as repression (Ponting *et al.*, 2009).

The functions of plant lncRNAs are being revealed. For instance, Swiezewski *et al.* (2009) reported that lncRNAs are involved in the regulation of flowering by altering the expression of *FLOWERING LOCUS C* (*FLC*) in Arabidopsis. Two lncRNAs that were transcribed from the antisense strand of *FLC* were designed as *COOLAIR*, and were found to be able to silence the expression of *FLC* (Swiezewski *et al.*, 2009). An intronic lncRNA, *COLDAIR*, can induce the epigenetic repression of *FLC* (Heo and Sung, 2011). Moreover, *APOLO* and *ASCO* have been reported to regulate root development in Arabidopsis (Ariel *et al.*, 2014). *PINOID* is a key regulator of polar auxin transport, and its expression can be up-regulated by the change in chromatin formation induced by the expression of the lncRNA *APOLO* (Ariel *et al.*, 2014). Nuclear Speckles RNA-binding proteins (NSRs) modulate the alternative splicing of a subset of genes involved in lateral root initiation, and this effect weakens when NSRs bind to *ASCO* (Bardou *et al.*, 2014). The lncRNA *HID1* mediates photomorphogenesis in Arabidopsis induced by red light (Wang *et al.*, 2014). The low expression of the lncRNA *LDMAR* was found to lead to male sterility under long-day conditions (Ding *et al.*, 2012), and *Enod40* has been shown to be associated with nodulation in legume plants (Campalans *et al.*, 2004). Under phosphate-deficient conditions, miR399 up-regulates the expression of Pi transport genes by cleaving *PHOSPHATE2* (*PHO2*), while *IPS1*, *At4*, and *Mt4* suppress the effect of miR399 by acting as mimics of *PHO2* (Burleigh and Harrison, 1997; Shin *et al.*, 2006; Franco-Zorrilla *et al.*, 2007). A *cis*-natural antisense lncRNA, *cis-NATPHO1;2*, positively regulates the expression of a protein that is critical for phosphate homeostasis in rice (Jabnoune *et al.*, 2013). Despite the progress made in these various studies, the functions and regulatory networks of lncRNAs in plants are less well known compared to those in mammals.

Advancements in high-throughput sequencing technology have allowed the accurate identification of lncRNAs in plants at the whole-genome level. LncRNAs have been identified in several model plant species, including Arabidopsis (Ben Amor *et al.*, 2009; Liu *et al.*, 2012; Zhu *et al.*, 2014), rice (Liu *et al.*, 2013; Xu *et al.*, 2016), maize (Li *et al.*, 2014), and poplar (Shuai *et al.*, 2014; Tian *et al.*, 2016). In contrast, little information is available regarding lncRNAs in the model legume *Medicago truncatula*. *Medicago truncatula* is an annual species that is widely used to study the functional genomics of legume plants because of its small diploid genome and amenability to transformation (Young *et al.*, 2011). Legume plants account for one-third of primary crop production worldwide, and often suffer from abiotic stresses (Benedito *et al.*, 2008). Emerging evidence indicates that lncRNAs play regulatory roles in responses to abiotic stress in plants (Liu *et al.*, 2012; Shuai *et al.*, 2014; Zhu *et al.*, 2014). Given the the great numbers and poorly known functions of lncRNAs compared to protein-coding genes, the functional characterization of lncRNAs has the potential to shed important light on their roles and could provide an effective tool to enhance yields of legume crops under unfavorable growth conditions (Liu and Zhu, 2014).

In our previous studies, we identified a number of osmotic- and salt stress-responsive lncRNAs in *M. truncatula*. By predicting the targets of lncRNAs, they were found to be implicated in numerous biological processes such as signal transduction and detoxification under abiotic stress (Wang *et al.*, 2015). Phosphorus (P) is one of the essential mineral nutrients for plant growth and development. Despite high total P content in soils, the amounts of inorganic phosphate (Pi) that are available to plants in many natural and agricultural ecosystems are often low, thus limiting plant growth and production (Hinsinger *et al.*, 2011; Nestler and Wissuwa, 2016). To cope with Pi deficiency in soils, plants have evolved numerous strategies at morphological, physiological, and molecular levels (Ding *et al.*, 2016; Nath and Tuteja, 2016; Dong *et al.*, 2017; Li *et al.*, 2017). Many protein-coding genes and microRNAs that are involved in sensing and responding to Pi deficiency have been identified (Plaxton and Tran, 2011). In contrast, few studies have specifically investigated the roles of lncRNAs in the regulation of physiological processes in response to Pi deficiency (Shin *et al.*, 2006; Franco-Zorrilla *et al.*, 2007; Jabnoune *et al.*, 2013; Xu *et al.*, 2016). In the present study, we identified a comprehensive set of Pi deficiency-responsive lncRNAs from *M. truncatula* by sequencing eight paired-end libraries, and functionally characterized three lncRNAs in response to Pi deficiency using the relevant mutants of *M. truncatula*.

## Materials and methods

### Plant material and stress treatments

*Medicago truncatula* Jemalong A17 is the model of legume plants, and its genome has been sequenced (Young *et al.*, 2011). *Medicago Tnt1* insertion mutants were generated based on *M. truncatula* R108 because of its relatively easy transformation (Tadege *et al.*, 2008). This mutant resource has been used in many studies of *Medicago*. Seeds of *M. truncatula* Jemalong A17, R108, and *Tnt1* insertion mutants were treated with concentrated sulfuric acid for 8 min, and then thoroughly rinsed with distilled water. After being chilled at 4 °C for 2 d, seeds were sown on 0.8% agar and germinated at 25 °C. When the radicals were about 2 cm long, the plants were transferred into plastic buckets filled with aerated nutrient solution under controlled conditions (26 °C day/22 °C night, and 14-h photoperiod). The composition of the full-strength nutrient solution was: 2.5 mM KNO_3_, 0.5 mM KH_2_PO_4_, 0.25 mM CaCl_2_, 1 mM MgSO_4,_ 100 µM Fe-Na-EDTA, 30 µM H_3_BO_3_, 5 µM MnSO_4_, 1 µM ZnSO_4_, 1 µM CuSO_4_, and 0.7 µM Na_2_MoO_4_, at pH 6.0.

Three-week-old seedlings of Jemalong A17 were transferred from the full-strength nutrient solution into a solution with reduced KH_2_PO_4_ concentration (1 µM) for either 12, 24, or 48 h. After exposure to the Pi-deficient media, samples were collected at the same time to discount any circadian effects. Leaf and root samples from 10 individual plants grown in control (CK, Pi-sufficient) and Pi-deficient (PD) media were harvested and dried at 80 °C and dry weights were determined once constant weight had been achieved. Three-week-old seedlings of R108 (the wild-type, WT) and *Tnt1* insertion mutants were treated using the same regime for 24 or 72 h. These materials were used to perform quantitative real-time PCR (qRT-PCR) and to determine P concentration, respectively.

### Construction of cDNA libraries and high-throughput sequencing

Seedlings of *M. truncatula* Jemalong A17 were used to construct libraries. Total RNA was extracted from leaves and roots exposed to Pi-sufficient and Pi-deficient media for 24 h using TRIzol (Invitrogen) according to the manufacturer’s protocols. Ribosome RNAs of RNA samples were removed using a Ribo-Zero™ Magnetic Kit (Epicentre). The strand-specific sequencing libraries were constructed following previously described protocols (Borodina *et al.*, 2011). Paired-end sequencing (2 × 100 bp) was performed on an Illumina Hiseq2000 sequencer. Two biological repeats were used in the construction of libraries.

### Read mapping and transcriptome assembly

The resulting directional paired-end reads were quality-checked with FastQC (http://www.bioinformatics.babraham.ac.uk/projects/fastqc/), and adapter contaminations and the low-quality tags in the raw data were discarded. Ribosome RNA data were also removed from the remaining data by alignment. Thereafter, the clean reads from the eight cDNA libraries were merged and mapped to the *M. truncatula* genome sequence (Mt4.0) using the spliced read aligner TopHat (Trapnell *et al.*, 2009). To construct transcriptomes, the mapped reads were assembled *de novo* using Cufflinks (Trapnell *et al.*, 2010). All the transcripts were required to have exons greater than 1 and longer than 200 bp in length.

### Identification of lncRNAs

The assembled transcripts were annotated using the Cuffcompare program from the Cufflinks package (Trapnell *et al.*, 2010). The known protein-coding transcripts were identified according to the annotation of *M. truncatula* genome sequences (Mt4.0). The remaining unknown transcripts were used to screen putative lncRNAs. Transcripts less than 200 nt in length and with less than three reads were first excluded. Then, the coding potentials of the remaining transcripts were calculated using the Coding Potential Calculator (CPC) and Coding-Non-Coding Index (CNCI) (Kong *et al.*, 2007; Sun *et al.*, 2013). A transcript with a CPC value less than –1 and a CNCI value lower than 0 was taken as being a non-coding one.

### Analysis of differential expression of lncRNAs

Expression levels of all transcripts, including those of putative lncRNAs and mRNAs, were quantified as fragments per kilobase of exon per million fragments mapped (FPKM) using the Cuffdiff program from the Cufflinks package (Trapnell *et al.*, 2010). Differential gene expression was determined by a *P*-value less than 0.05.

### Prediction of putative *cis*- and *trans*-targets of lncRNAs

The transcription of lncRNAs has been implicated in the regulation of the expression of genes in close genomic proximity (*cis*-acting regulation) and in the targeting of distant genes (*trans*-acting regulation) via multiple mechanisms (Ponting *et al.*, 2009; Kornienko *et al.*, 2013). Many studies have demonstrated that one important function of lncRNAs is to regulate the expression of neighboring protein-coding genes via epigenetic modification and/or transcriptional co-activation/repression (Rinn *et al.*, 2007; Yu *et al.*, 2008; Mercer *et al.*, 2009). Therefore, the analyses were performed of genomic co-locations (<10 kb) of the lncRNAs and mRNAs according to previously described methods (Liao *et al.*, 2011). In addition, the formation of near-complementary lncRNA–target duplexes is also an important way to regulate the expression of their *trans*-targets (Chekanova *et al.*, 2007; Ponting *et al.*, 2009). The *trans*-targets of lncRNAs were predicted by the complementarity of lncRNAs and their targets with expression markedly different under Pi-deficient conditions using RIsearch (Wenzel *et al.*, 2012). Finally, those putative *cis*- and *trans*-targets of lncRNAs were analysed using gene ontology (GO) (Ashburner *et al.*, 2000), and GO terms were considered to be enriched when the *P* value was less than 0.05 using Blast2GO (Conesa *et al.*, 2005).

### GFP analyses

Expression vectors of 35S:*PDIL2*, 35S:*PDIL3*, and 35S:*Medtr1g074930*:*GFP* were constructed for transient transformation of *Nicotiana benthamiana* following the protocols described by Franco-Zorrilla *et al.* (2007). Either *PDIL2* or *PDIL3* was co-transformed into leaves of *N. benthamiana* with Medtr1g074930:GFP by agroinfiltration. In this assay, the intensity of GFP (green fluorescent protein) indicates the expression level of Medtr1g074930. Images were taken under identical conditions using a fluorescence microscope (Nikon Eclipse Ti). At least five images were used to analyse the relative intensity of GFP using the ImageJ software.

### Quantitative real-time PCR

Total RNA was isolated using RNAiso Plus reagent (TaKaRa) and treated with RNase-free DNase I (Promega). About 0.5 μg RNA was reverse-transcribed into first-strand cDNA with a PrimeScript^®^ RT reagent Kit (TaKaRa). Quantitative real-time PCR (qRT-PCR) was performed using an ABI Stepone Plus Instrument. Gene-specific primers and internal control primers are listed in Supplementary Table S1 at *JXB* online.

All qRT-PCR reactions were performed in triplicates for each cDNA sample with an annealing temperature of 57 °C and a total of 40 cycles of amplification. The relative expression level was calculated by the comparative C_T_ method (Livak and Schmittgen, 2001).

### Identification and confirmation of homozygotic mutants

Generation of the *M. truncatula Tnt1* insertional mutants was described previously (Tadege *et al.*, 2008). Mutants were identified by aligning lncRNA sequences with the *M. truncatula* mutant database (http://medicago-mutant.noble.org/mutant/blast/blast.php).

Genomic DNA from mutants was isolated using a Plant Genomic DNA Kit (Tiangen). Homozygotes were identified using two sets of PCR primers as follows: for *pdil1-1* (*PDIL1*-F+*TNT1*-R and *PDIL1*-F+*PDIL1*-R), for *pdil1-2* (*PDIL1*-F+*TNT1*-F and *PDIL1*-F+*PDIL1*-R), for *pdil2* (*PDIL2*-F+*TNT1*-F and *PDIL2*-F+*PDIL2*-R), and for *pdil3* (*PDIL3*-F+*TNT1*-R and *PDIL3*-F+*PDIL3*-R). Primers are listed in Supplementary Table S1. If the first reaction was positive and the second reaction was negative, the mutant was a homozygote. The expression levels of *PDILs* in WT and mutants were monitored by RT-PCR. Seeds of homozygotes were harvested and used in this study.

### Determination of P concentration in leaves and roots

Leaf and root samples exposed to Pi-sufficient and Pi-deficient media were harvested and dried at 80 °C to constant weight. A mixture of 50 mg of dry material, 5 ml of nitric acid, and 2 ml of hydrogen peroxide was placed in digestion tubes, and then samples were digested using a microwave system (MARS, CEM). After diluting and filtering, P concentrations were measured using an ICP-AES (Thermo).

### Statistical analysis

Data were analysed using SPSS statistics 17.0. One-way ANOVA analysis with Duncan tests was performed for multiple comparisons, and *t*-tests were performed to test for significant differences between the two groups of data in this study.

### Accession numbers

RNA-seq data are available in the Sequence Read Archive database (https://www.ncbi.nlm.nih.gov/sra) under accession numbers SRR1523070 and SRR3938213 for CK-L, SRR1523071 and SRR3938216 for CK-R, SRR1536246 and SRR3938252 for PD-L, and SRR1536247 and SRR3938256 for PD-R.

## Results

### Plant P concentrations

In this study, 3-week-old seedlings of *M. truncatula* previously grown in Pi-sufficient medium (0.5 mM Pi) were exposed to Pi-deficient (1 µM Pi) medium for 0, 12, 24 or 48 h. We sampled both leaves and roots for determination of biomass and P concentrations. As shown in Table 1, exposure to Pi-deficient medium for 24 h led to significant reductions in dry biomass of roots, and in P concentrations in leaves and roots. In contrast, foliar dry biomass was relatively constant after treatment with Pi deficiency for 24 h (Table 1). These results suggested that the plants exposed to P-deficient medium for 24 h were at an early stage of stress response, and that plants may actively mobilize genes and regulatory networks to cope with Pi deficiency. Therefore, we sampled roots and leaves at this point to construct cDNA libraries in order to identify Pi deficiency-responsive lncRNAs.

**Table 1.** Time-dependent changes in plant growth and P concentrations in leaves and roots of *M. truncatula* Jemalong A17 seedlings in response to Pi deficiency

Treatment time (h)	Dried biomass (mg plant^−1^)	P concentration (mg g^−1^ DW)	P content (mg plant^−1^)
Leaves			
0	35.700 ± 1.159a	10.645 ± 0.486a	0.380 ± 0.017a
12	33.180 ± 0.961a	10.502 ± 0.328a	0.348 ± 0.011a
24	32.980 ± 0.873a	8.806 ± 0.191b	0.290 ± 0.006b
48	29.080 ± 1.161b	7.642 ± 0.188c	0.222 ± 0.005c
Roots			
0	16.360 ± 0.923a	7.645 ± 0.487a	0.125 ± 0.008a
12	15.570 ± 0.522a	7.253 ± 0.241a	0.113 ± 0.004a
24	13.530 ± 0.614b	5.873 ± 0.080b	0.080 ± 0.001b
48	12.270 ± 0.693b	4.909 ± 0.213c	0.060 ± 0.003c

Data are the means ±SE. For dried biomass, there were 10 replicates. For P concentration and content, there were three replicates. Different letters indicate significant differences among treatments (*P*<0.05).

### RNA-seq of eight cDNA libraries

Leaf and root samples of *M. truncatula* Jemalong A17 exposed to Pi-deficient and Pi-sufficient media for 24 h were used to construct cDNA libraries and were sequenced by an Illumina-Solexa sequencer. Two biological repeats were used to construct the libraries. High-throughput RNA-sequencing (RNA-seq) of the eight libraries led to generation of 741 571 940 clean reads and 111.24 G clean bases (Table 2). A quality score (Q) for each base in the reads was calculated by a phred-like algorithm using FastQC (Ewing and Green, 1998), and the results showed that the data were highly credible (Supplementary Fig. S1).

**Table 2.** Statistical data of the RNA-seq reads for the eight libraries constructed from leaves and roots of *M. truncatula* Jemalong A17 exposed to control (CK, Pi-sufficient) and Pi-deficient (PD) media

	CK		PD	
	Leaves	Roots	Leaves	Roots
Raw reads	95 999 176 + 77 703 184	93 999 446 + 92 619 970	164 698 304 + 91 928 280	89 588 642 + 78 257 344
Raw bases	14.40 G + 11.66 G	14.10 G + 13.89 G	24.70 G + 13.79 G	13.44 G + 11.74 G
Clean reads	87 897 824 + 75 538 710	87 087 836 + 90 435 180	153 503 868 + 89 656 196	81 349 080 + 76 103 276
Clean bases	13.18 G + 11.33 G	13.06 G + 13.57 G	23.03 G + 13.45 G	12.20 G + 11.42 G
Unique lncRNAs	7381	9454	7213	9614

### Identification and characterization of lncRNAs

After the clean reads were mapped, assembled, and annotated according to the *M. truncatula* genome sequence (Mt4.0) using the TopHat and Cufflinks packages (Trapnell *et al.*, 2009, 2010), the known protein-coding RNAs were first identified. The remaining reads were then filtered by length and coding potential, such that transcripts smaller than 200 bp were excluded, and the transcripts with CPC more than –1 and CNCI greater than 0 were removed. The remaining transcripts were considered as putative lncRNAs.

**Fig. 1.**
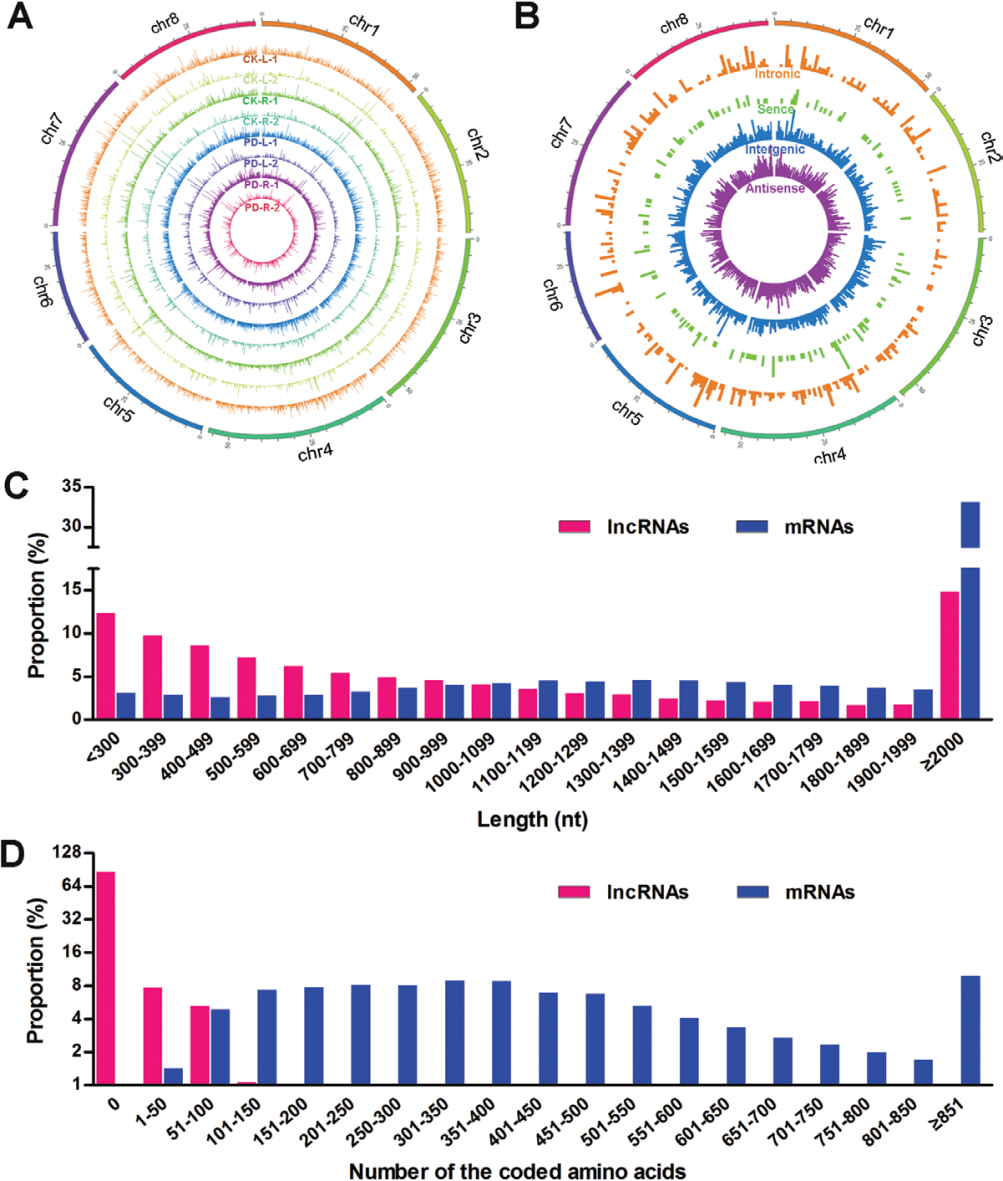
Characteristics of *M. truncatula* lncRNAs. (A) The expression level of lncRNAs (log_10_ FPKM) along the eight *M. truncatula* chromosomes. The eight concentric rings correspond to different samples: two control (Pi-sufficient) samples of leaves (CK-L), two control samples of roots (CK-R), two Pi-deficient samples of leaves (PD-L), and two Pi-deficient samples of roots (PD-R). (B) Distribution of different types of lncRNAs. The intronic, sense, intergenic, and antisense lncRNAs are represented by the different concentric rings from outer to inner, according to the loci of lncRNAs in the genome. (C) Length distribution of lncRNAs and mRNAs. (D) Length distribution of amino acids coded by lncRNAs and mRNAs.

The number of unique lncRNAs identified in the leaves and roots of *M. truncatula* Jemalong A17 exposed to Pi-deficient and Pi-sufficient media for 24 h is shown in Table 2. In total, we obtained 10 785 unique lncRNAs, and their loci are shown in Supplementary Table S2. Using the Circos program (Krzywinski *et al.*, 2009), the expressional distribution of lncRNAs from the eight libraries were drawn along eight chromosomes, and they exhibited no obvious preference for locations (Fig. 1A). In terms of the loci in the genome, 4811 intergenic, 599 intronic, 161 sense, and 5214 antisense lncRNAs were distinguished (Fig. 1B). Compared with protein-coding genes, the length of lncRNAs was much shorter (Fig. 1C). Among the lncRNAs identified in this study, 85.7% cannot code for proteins, and the remaining lncRNAs only coded for proteins with short chains of amino acids (Fig. 1D).

### Response of lncRNAs to Pi deficiency

**Fig. 2.**
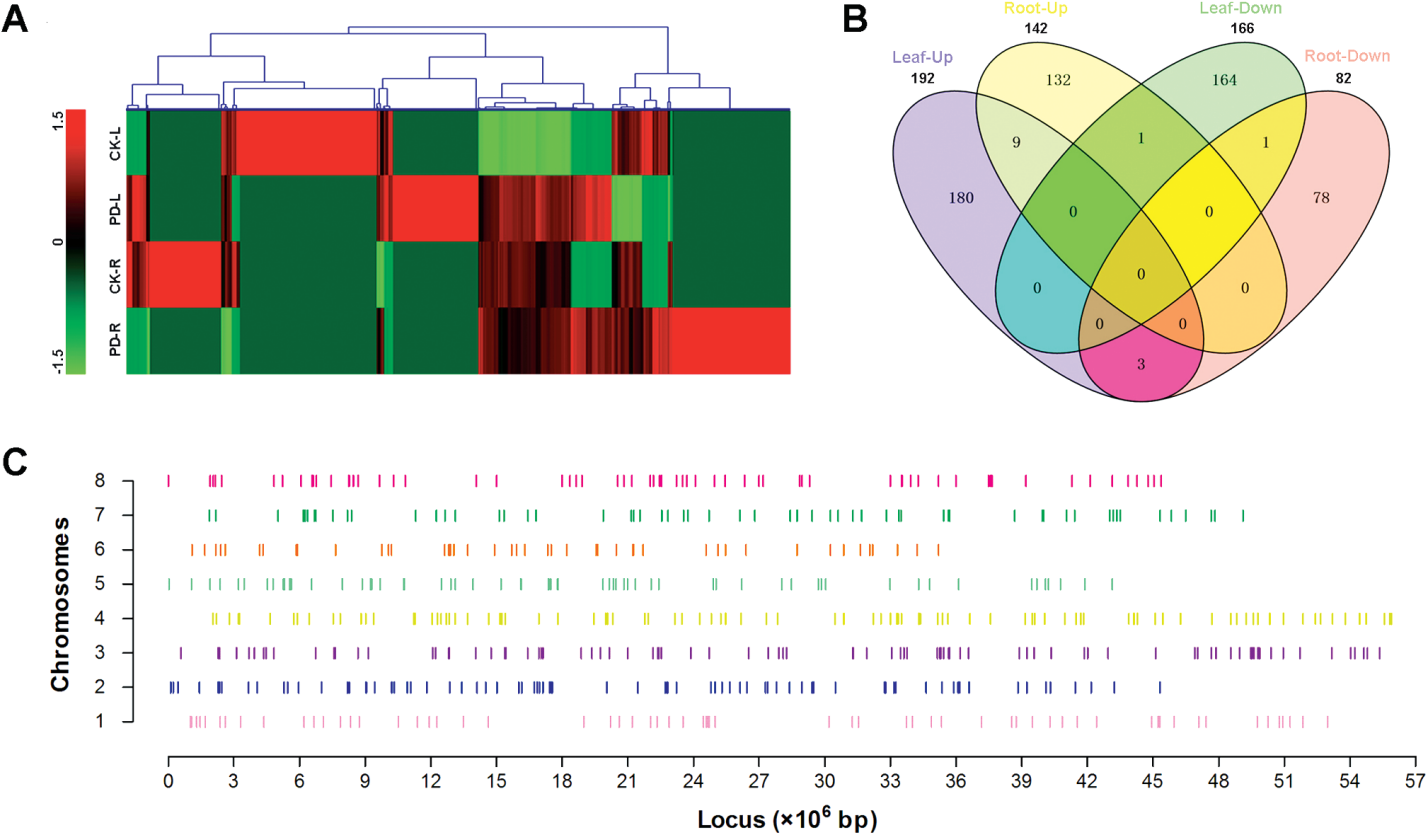
Heatmap (A), Venn diagram (B), and locus (C) of Pi-deficiency lncRNAs. The gene tree in (A) was drawn by the method of Hierarchical Clustering, and the *Z*-values of log_2_ FPKM were used in this analysis. CK, control (Pi-sufficient); PD, Pi-deficient; L, leaves; R, roots. In (C) the short vertical lines indicate the loci of Pi-deficiency lncRNAs in eight chromosomes.

To identify the Pi deficiency-responsive lncRNAs, we calculated and compared the FPKM values in the four treatments (eight libraries) (Fig. 2A). Transcript levels of 358 lncRNAs in the leaves and 224 lncRNAs in the roots were altered by Pi deficiency (Supplementary Table S3), and they were designated as *P*hosphate *D*eficiency-*I*nduced *L*ncRNAs (*PDIL*s). LncRNAs with expression up- or down-regulated by Pi deficiency in both leaves and roots are shown in the Venn diagram in Fig. 2B. We further grouped the Pi deficiency-responsive lncRNAs into those that were common or specific. For example, nine lncRNAs in both leaves and roots were up-regulated in response to Pi deficiency, while one lncRNA was down-regulated in both tissues. The Pi deficiency-responsive lncRNAs were found to be located across all the chromosomes, with the most abundant lncRNAs on Chromosome 4 (Fig. 2C).

### Functional analysis of Pi deficiency-responsive lncRNAs

Previous studies demonstrated that the genes encoding lncRNAs were preferentially located next to protein-coding genes, and that they regulated the expression of the *cis*- or *trans*-targets by forming near-complementary lncRNA–target duplexes (Rinn *et al.*, 2007; Yu *et al.*, 2008; Mercer *et al.*, 2009; Kornienko *et al.*, 2013). To examine the potential roles of Pi deficiency-responsive lncRNAs, we analysed the GO terms of putative targets of the lncRNAs.

**Fig. 3.**
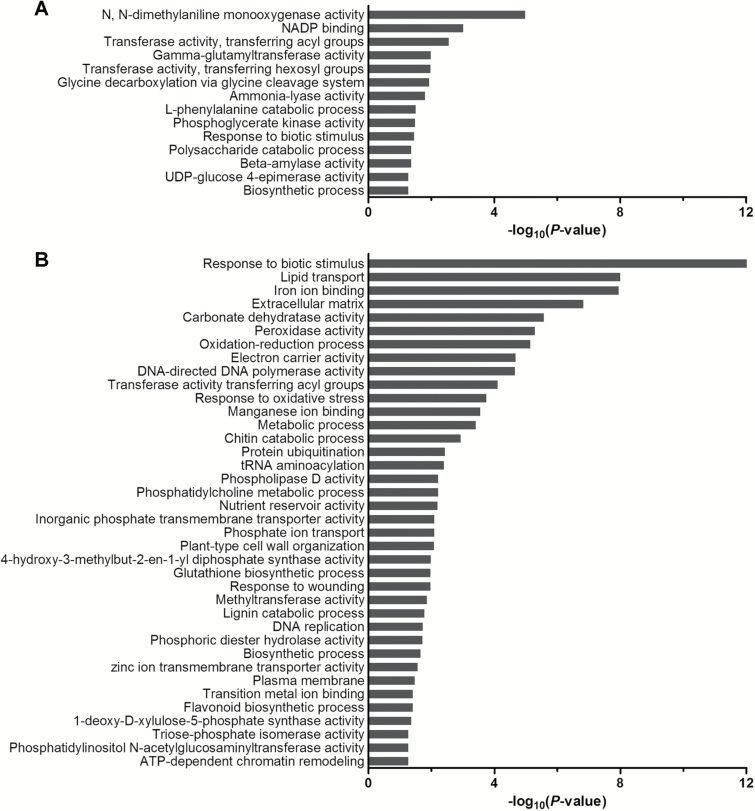
GO enhancements of putative targets of lncRNAs in leaves (A) and roots (B) of *M. truncatula* under Pi deficiency. The reliability is calculated by –log_10_(*P*-value). More detailed information is given in Supplementary Tables S4 and S5.

A significant enrichment of putative targets in 14 and 38 GO terms was detected in leaf and root samples, respectively, under the Pi-deficient conditions (Fig. 3, Supplementary Tables S4, S5). The higher number of enriched GO terms in the roots may suggest that they are more sensitive to Pi deficiency than the leaves. The findings also suggest that the Pi deficiency-responsive lncRNAs may regulate the expression of genes involved in many biological processes, including those of signal transduction, energy synthesis, and detoxification. Moreover, some lncRNAs may directly regulate the transport of phosphate (GO: 0006817, Phosphate transport).

### Functional characterization of *PDIL1*, *PDIL2*, and *PDIL3*

**Fig. 4.**
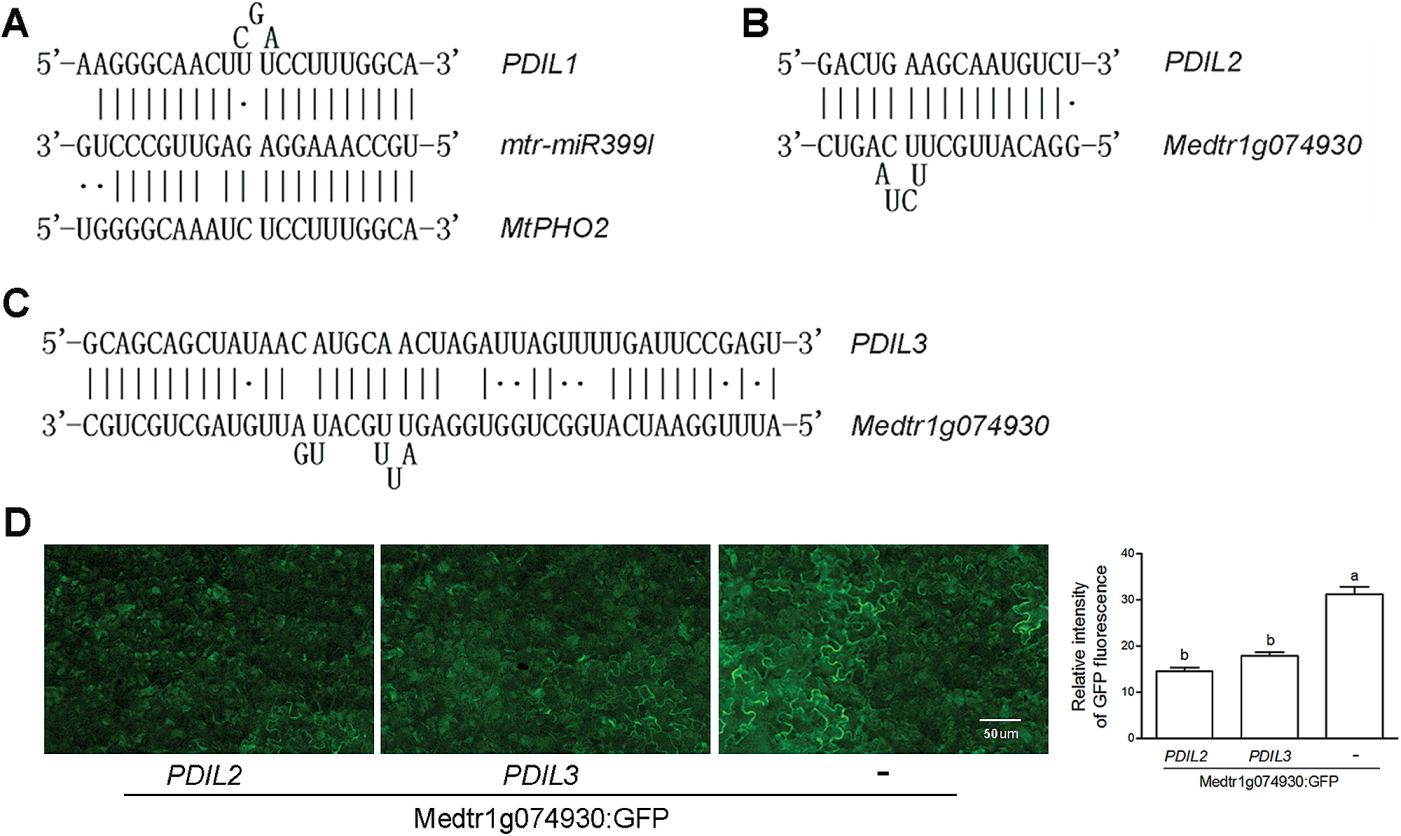
Regulation of targets by *PDIL*s. (A) Sequence complementary positions of mtr-miR399l, *PDIL1*, and *MtPHO2*. (B) Sequence complementary positions of *PDIL2* and its target *Medtr1g074930*. (C) Sequence complementary positions of *PDIL3* and its target *Medtr1g074930*. Perfect base pairing is depicted with a vertical line, while G–U wobble pairing is marked with a point. Alignments of full sequences between *PDIL1* and *Mt4*, and between *PDIL2*, *PDIL3*, and *Medtr1g074930* are shown in Supplementary Fig. S2. (D) Transient expression assays in *N. benthamiana*. Expression of Medtr1g074930:GFP was monitored by fluorescence microscopy. Control agroinfiltration using a strain with an empty vector is indicated by ‘–’.

We identified that *PDIL1* was an *Mt4*-like lncRNA by sequence alignment (Supplementary Fig. S2A). Moreover, both *PDIL1* and *MtPHO2* (*Medtr2g013650*) are predicted to be targeted by mtr-miR399l in *M. truncatula* (Fig. 4A). Therefore, *PDIL1* can competitively inhibit *MtPHO2* degradation as a target mimic of miR399. In addition, *PDIL2* and *PDIL3* may directly repress the expression of the Pi transporter gene *Medtr1g074930* by complementary binding (Fig. 4B–D, Supplementary Figs S2B and S3).

By means of expression in *N. benthamiana*, Franco-Zorrilla *et al.* (2007) reported that *Mt4*-like lncRNA was involved in the regulation of a Pi transporter gene. We used the same method to test whether *PDIL2* and *PDIL3* play a role in the regulation of the Pi transporter gene *Medtr1g074930*. As shown in Fig. 4D, expression of *PDIL2* and *PDIL3* in *N. benthamiana* led to a reduction in the relative intensity of Medtr1g074930:GFP.

**Fig. 5.**
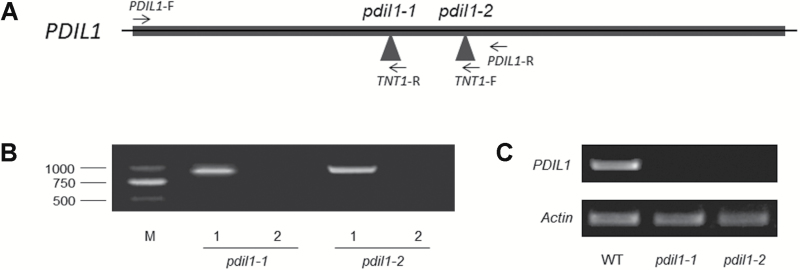
Identification and confirmation of *pdil1* mutants. (A) The insertional positions of *Tnt1* in *pdil1* mutations. Triangles indicate insertional positions of *Tnt1*. The locations of primers used for identification of homozygotes are labelled. (B) Identification of homozygotic *pdil1* mutants. Homozygotes were identified using two sets of PCR primers as follows: for *pdil1-1*, *PDIL1*-F+*TNT1*-R in lane 1 and *PDIL1*-F+*PDIL1*-R in lane 2; for *pdil1-2*, *PDIL1*-F+*TNT1*-F in lane 1 and *PDIL1*-F+*PDIL1*-R in lane 2. (C) The expression levels of *PDIL1* in *pdil1* mutants. Primers are listed in Supplementary Table S1.

To further study the function of the Pi deficiency-responsive lncRNAs, mutants of these lncRNAs were identified from the *M. truncatula* mutant database (https://medicago-mutant.noble.org/mutant/). NF19212 (*pdil1-1*) and NF8919 (*pdil1-2*) were identified as two mutants for *PDIL1* (Fig. 5A), and NF7430 (*pdil2*) and NF21369 (*pdil3*) were identified as mutants for *PDIL2* and *PDIL3*, respectively (Supplementary Fig. S4A). Homozygotes of these mutants were validated using PCR (Fig. 5B, Supplementary Fig. S4B). We did not detect the expression of these lncRNAs in the mutants by RT-PCR (Fig. 5C, Supplementary Fig. S4C).

**Fig. 6.**
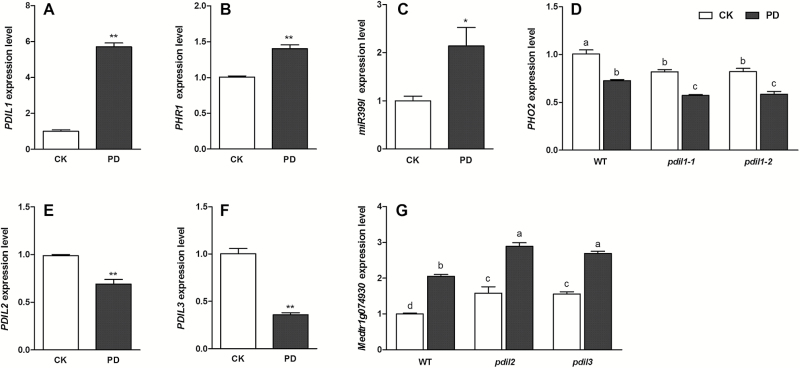
The expression levels of *PDIL1* (A), *PHR1* (B), miR399l (C), *PHO2* (D), *PDIL2* (E), *PDIL3* (F), and *Medtr1g074930* (G) in roots of wild-type (WT) *M. truncatula* under Pi-sufficient (control, CK) or Pi-deficient (PD) conditions. The expression levels of *PHO2* and *Medtr1g074930* were detected in *pdil* mutants. Data with ‘*’ or different letters indicate significant differences (*P*<0.05) between treatments and control.

Under Pi-deficient conditions, the expression of a MYB transcription factor gene *MtPHR1* (*Phosphate Starvation Response 1*) was up-regulated in roots, and mtr-miR399l was induced by Pi deficiency (Bari *et al.*, 2006;). *MtPHO2* was suppressed as the target of miR399, and it can minimize degradation of Pi transporters in the post-translational processes (Huang *et al.*, 2013). At the same time, *PDIL1* was positively regulated to suppress the effect of miR399, thus avoiding the superfluous accumulation of P. Expression-levels of *PDIL1*, *PHR1*, and *miR3991* (Fig. 6A–C) in WT plants were up-regulated by Pi deficiency. In contrast, expression of *PHO2* in WT plants was down-regulated by Pi deficiency (Fig. 6D). Moreover, the mutation of *PDIL1* led to a lower expression of *MtPHO2* under both Pi-sufficient and Pi-deficient conditions (Fig. 6D). The expression of *PDIL2* and *PDIL3* (Fig. 6E, F) in WT plants was down-regulated upon exposure to Pi-deficient medium, while expression of a Pi transporter gene *Medtr1g074930* was up-regulated by the Pi deficiency (Fig. 6G). The expression-levels of *Medtr1g074930* in *pdil2* and *pdil3* mutants were greater than in WT plants under both Pi-sufficient and Pi-deficient conditions (Fig. 6G).

The mutants did not show any visible phenotypic differences relative to their WT counterparts, and exhibited similar biomass to WT plants after exposure to Pi-deficient medium for 3 d. In the Pi-sufficient medium, no significant differences in P concentrations of leaves and roots were detected between *pdil1* mutants and WT plants (Table 3). P concentrations in the roots of *pdil1* mutants and WT plants were comparable after exposure to Pi-deficient medium. However, P concentrations in leaves of *pdil1* mutants were significantly higher than in those of WT plants under Pi-deficient conditions (Table 3). Similar to *pdil1*, *pdil2* and *pdil3* mutants also exhibited greater capability to acquire P, as evidenced by higher P concentrations in their shoots than in WT plants.

**Table 3.** Effects of Pi deficiency on P concentrations in leaves and roots of the wild-type (WT) and different mutants for plants exposed to control (CK, Pi-sufficient) and Pi-deficient (PD) media

	Leaf P concentration (mg g^−1^ DW)		Root P concentration (mg g^−1^ DW)	
	CK	PD	CK	PD
WT	10.265 ± 0.283	7.353 ± 0.154	7.123 ± 0.385	3.972 ± 0.183
*pdil1-1*	10.499 ± 0.519	8.638 ± 0.161*	7.268 ± 0.249	4.181 ± 0.254
*pdil1-2*	10.293 ± 0.400	8.758 ± 0.424*	7.376 ± 0.139	4.051 ± 0.131
*pdil2*	12.630 ± 0.447*	8.788 ± 0.535*	8.885 ± 0.592*	4.286 ± 0.178
*pdil3*	12.360 ± 0.930*	9.568 ± 0.503**	8.931 ± 0.853*	4.326 ± 0.244

Data are the means ±SE (*n*=3). *P<0.05, **P<0.01: significant differences between treatments and control.

## Discussion

Despite the greater quantity of lncRNAs than of protein-coding genes in the genomes, little is known about the functions of lncRNAs in plants. Functional characterization of lncRNAs and deciphering their regulatory mechanisms is crucial to advance our knowledge. Identification of lncRNAs at the whole-genome level has been conducted in several plant species by high-throughput sequencing (Ben Amor *et al.*, 2009; Liu *et al.*, 2012, 2013; Li *et al.*, 2014; Shuai *et al.*, 2014; Zhu *et al.*, 2014; Tian *et al.*, 2016; Xu *et al.*, 2016); however, the methods used in some of the studies have not been comprehensive. For example, lncRNAs without poly(A) were not included and only the intergenic lncRNAs were identified (Shuai *et al.*, 2014). In addition, most of the libraries used for sequencing of lncRNAs were without biological repeats, and the functions of lncRNAs have often been predicted by bioinformatics approaches without experimental data (Zhang *et al.*, 2014).

In the present study, we identified all the sense, antisense, bidirectional, intronic, and intergenic lncRNAs, and included the lncRNAs both with and without poly(A) (Fig. 1B). Moreover, we detected low-expressional and tissue-specific lncRNAs using large amounts of data from leaf and root samples. More importantly, two biological repeats of libraries and highly credible data (Supplementary Fig. S1) ensured that our transcriptomic analysis is highly reproducible and reliable, and this was validated by qRT-PCR (Fig. 6A, E, F). We further characterized the functions of three Pi deficiency-responsive lncRNAs using their *Tnt1* mutants. To the best of our knowledge, our results identified, for the first time, the most comprehensive Pi deficiency-responsive lncRNAs at the whole-genome level in the higher plants.

Phosphorus is a key component of many macromolecules and ATP in plant cells, and plays important roles in enzymatic reactions and signal transduction (Chiou and Lin, 2011; Dai *et al.*, 2016). Despite high amounts of total P in soils, the inorganic phosphate (Pi) that can be directly acquired by plants is low, and globally approximately 70% of cultivated land suffers from Pi deficiency (Plaxton and Tran, 2011; Li *et al.*, 2016). To cope with deficiency, plants have evolved numerous strategies to acquire Pi from soils. The involvements of *SIZ1*, *PHR1*, miR399, and *PHO2* in the regulation of Pi acquisition have been well established in plants (Bari *et al.*, 2006; Chiou and Lin, 2011). For instance, the expression of miR399 is positively regulated by the transcription factor PHR1 that is induced by Pi deficiency. *PHO2*, a target of miR399, encodes a ubiquitin-conjugating E2 enzyme that can degrade Pi transporters, such that cleavage of *PHO2* confers greater acquisition of Pi by roots under Pi-deficient conditions (Bari *et al.*, 2006; Huang *et al.*, 2013; Park *et al.*, 2014). There have been reports implying that lncRNAs participate in signal transduction of Pi in plants. For example, Pi starvation markedly induced expression of *Mt4*, and *At4* and *IPS1* were identified as two *Mt4*-like lncRNAs in Arabidopsis (Bazin and Bailey-Serres, 2015). *At4* and *IPS1* share a conserved motif, showing partial complementarity with miR399. Thus, they can competitively bind to miR399 as target mimics to protect *PHO2* transcripts from degradation by miR399 (Shin *et al.*, 2006; Franco-Zorrilla *et al.*, 2007). The functions of *At4* and *IPS1* have been characterized by overexpression lines and mutants (Shin *et al.*, 2006; Franco-Zorrilla *et al.*, 2007), but the function of *Mt4* in the regulation of Pi homeostasis has not been validated experimentally using genetic material in *M. truncatula*.

Here, we identified a Pi deficiency-responsive lncRNA, *PDIL1*, that is a close paralog of *Mt4*. To characterize the function of *PDIL1*, we obtained two mutants from the *M. truncatula* mutant database. The mutation of *PDIL1* potentiated the negative regulation of miR399 to *PHO2*, leading to a lower expression of *PHO2* in the *pdil1* and *pdil2* mutants than that of WT plants in Pi-sufficient medium (Fig. 6D). Moreover, the *PHO2* transcripts were reduced by enhanced expression of miR399 in both WT and mutants under Pi-deficient conditions, while a lower expression level of *PHO2* was observed in roots of mutants than WT plants (Fig. 6D). *PHO2* encodes a ubiquitin-conjugating enzyme, UBC24, which mediates degradation of high-affinity Pi transporters. It is conceivable that the activity of Pi transporters in *pdil1* is greater than that in the WT plants, thus conferring the *pdil1* mutant greater ability to acquire Pi. Our results showed that P concentrations in roots of *pdil1* were comparable to those in WT plants. However, mutation of *pdil1* rendered a greater accumulation of P in shoots under Pi-deficient conditions (Table 3). In Arabidopsis, overexpression of *At4* and *IPS1* resulted in a decrease in shoot P concentration in Pi-sufficient medium, and mutation of *At4* increased P concentrations in shoots under Pi-deficient conditions (Shin *et al.*, 2006; Franco-Zorrilla *et al.*, 2007). In the present study, we found that mutation of *PDIL* led to an increase in P concentrations in leaves (Table 3). By contrast, no significant differences in P concentrations in roots between the WT and the *pdil1* mutants were observed (Table 3). This observation may suggest that P taken up by roots is translocated preferentially into shoots to participate in important biological processes such as photophosphorylation. In Pi-sufficient medium, we did not observe the greater accumulation of P in the *pdli1* mutants. This suggests that *PDIL1* may not directly regulate the Pi transporters; instead, it may indirectly control the Pi transport by PHO2 via unknown mechanisms, thus allowing the plant to maintain a constant P concentration under Pi-sufficient conditions. In addition, we found that Pi deficiency down-regulated expression of *PDIL2* and *PDIL3* (Fig. 6E, F). We further demonstrated that expression of *PDIL2* and *PDIL3* in *N. benthamiana* suppressed expression of the Pi transporter gene *Medtr1g074930* (Fig. 4B–D). Mutations of the two lncRNAs resulted in a greater ability to acquire P by the mutants. In Pi-sufficient medium, accumulation of P in both leaves and roots of *pdil2* and *pdil3* was greater than that of the WT. However, P contents in the leaves of the *pdil2* and *pdil3* mutants under Pi-deficient conditions were higher than those in WT plants (Table 3). A similar explanation to that of the *pdil1* mutant may be used to account for the difference. Functional elucidation of these lncRNAs highlights their important roles in the regulation of Pi homeostasis in plants.

**Fig. 7.**
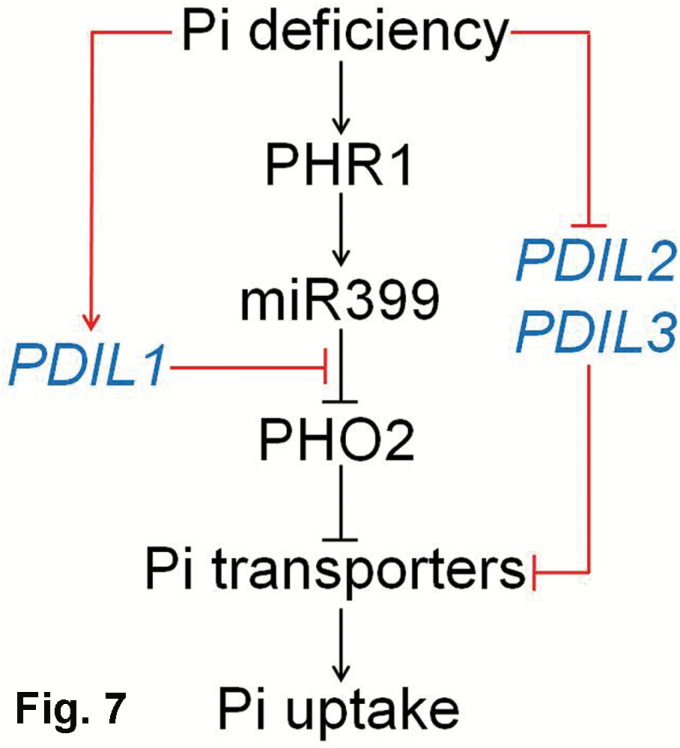
A model for Pi signaling involving PHR1, miR399, PHO2, and three Pi deficiency-responsive lncRNAs (*PDIL1, PDIL2*, and *PDIL3*) in *M*. *truncatula*. The red lines represent the pathways identified in the present study. Arrows denote positive effects, whereas lines ending with a short bar indicate negative effects.

Based on our results, we propose a model for the involvement of lncRNAs in the regulation of a Pi-deficient signaling pathway (Fig. 7). In this model, the *PHR1*–miR399–*PHO2* pathway has been established to play a central role in the regulation of P acquisition. Upon exposure to Pi-deficient medium, the MYB transcription factor genes of *PHR1* and miR399 are up-regulated consecutively. The expression of *PHO2*, a target of miR399, is suppressed by miR399 under Pi- deficient conditions. PHO2 is involved in the degradation of Pi transporters by the ubiquitination pathway, thus leading to an increase in P acquisition by up-regulating the activity of Pi transporters. The two molecular pathways converge to form Pi-dependent signaling cascades. It is predicated that *PDIL2* and *PDIL3* negatively regulate the expression of the Pi transporter gene *Medtr1g074930*. The down-regulation of *PDIL2* and *PDIL3* evoked by Pi deficiency up-regulates the transcript of *Medtr1g074930*. Both changes in the *PHR1*–miR399–*PHO2* and *PDIL2*/*PDIL3* pathways can enhance P acquisition under Pi-deficient conditions. In contrast, the *Mt4*-like lncRNAs, including *PDIL1*, are induced by Pi deficiency, and they negatively regulate P acquisition. *PDIL1* shares a conserved motif with *PHO2* that can be identified by miR399 and *PDIL1*. Therefore, *PDIL1* can competitively bind to miR399 as a target mimic to inhibit *PHO2*, leading to a negative regulation of P acquisition. The two directional efforts form a regulatory loop to maintain P homeostasis, thus allowing plants to perform vari- ous physiological processes at an appropriate Pi concentration.

In conclusion, we obtained 111.24 G clean sequence data from sequencing eight paired-end libraries, and identified 10 785 lncRNAs from the legume model plant *M. truncatula*. By GO enrichment of the targets of Pi deficiency-responsive lncRNAs, we showed that these lncRNAs were involved in the regulation of signal transduction, energy synthesis, detoxification, and phosphate transport. We further demonstrated that the lncRNAs *PDIL1-3* were involved in the regulation of the Pi-deficiency network and Pi transport. These results provide valuable information for our understanding of the functions of lncRNAs in response to Pi deficiency.

## Supplementary data

Supplementary data are available at *JXB* online.

Table S1. Sequences of primers used in this study.

Table S2. All putative lncRNAs identified in this study.

Table S3. Information relating to Pi deficiency-responsive lncRNAs.

Table S4. GO enhancements of the putative targets of lncRNAs in leaves under P deficiency.

Table S5. GO enhancements of the putative targets of lncRNAs in roots under P deficiency.

Fig. S1. Quality score values of RNA-seq from eight samples.

Fig. S2. Alignments of full sequences between *PDIL1* and *Mt4*, and between *PDIL2*, *PDIL3*, and *Medtr1g074930*.

Fig. S3. Alignments of protein sequences between Medtr1g074930 and other phosphate transporters of Arabidopsis and rice.

Fig. S4. Identification and confirmation of *pdil2* and *pdil3* mutants.

## Supplementary Material

supplementary_table_S1_S4_S5_Figures_S1_S4

supplementary_table_S2

supplementary_table_S3
