# Supplementary material for: Novel phosphate deficiency-responsive long non-coding RNAs in the legume model plant Medicago truncatula
Source: J Exp Bot. 2017 Nov 20;68(21-22):5937–48. doi: 10.1093/jxb/erx384 (PMC5854128; doi:10.1093/jxb/erx384)

Novel phosphate deficiency-responsive long non-coding RNAs in a legume model plant *Medicago truncatula*

Tianzuo Wang, Mingui Zhao, Xiuxiu Zhang, Min Liu, Chenge Yang, Yuhui Chen, Rujin Chen, Jiangqi Wen, Kirankumar S. Mysore, Wen-Hao Zhang

Table S1 Sequences of primes using in this study.

For identification of homozygous mutants:

Primers	Sequences (5'-3')
PDIL1-I-F	ATGGAACCACATGGGATA
PDIL1-I-R	CACAACAACACACAAACAA
PDIL2-I-F	GGTAACTCATTTGGTGTTG
PDIL2-I-R	TTCCATGCATACTCTATAGG
PDIL3-I-F	GCGTGGCTTGTTACTTGA
PDIL3-I-R	AAATCTTCTGTTGGCATA
TNT1-I-F	ACAGTGCTACCTCCTCTGGATG
TNT1-I-R	CAGTGAACGAGCAGAACCTGTG

For RT-qRCR or RT-PCR:

Primers	Sequences (5'-3')
PDIL1-F	ATGGAACCACATGGGATA
PDIL1-R	CACAACAACACACAAACAA
PDIL2-F	TGTTCTTAGTTTGGCTCA
PDIL2-R	CACATTCGTATTTACAAGTC
PDIL3-F	GACCCCAGACATATTAGC
PDIL3-R	AAATCTTCTGTTGGCATA
PHR1-F	CTTCCTTCATCGTTGTC
PHR1-R	TTTTCCACATAAGTCGG
mtr-miR399l-F	AACAATAGGGCACCTCTCT
mtr-miR399l-R	AGCCACAGGGCAACTCTC
PHO2-F	GTTGGTGCGTCAGGGACA
PHO2-R	GGTTCATTGGGATACTCG
Mt1g074930-F	GGTATCAAGAACTCCCTCA
Mt1g074930-R	TCAATAGCCTCAGCATCG
Actin-F	ACGAGCGTTTCAGATG
Actin-R	ACCTCCGATCCAGACA

Table S2 All putative lncRNAs identified in this study. (Excel file)

Table S3 The information of Pi deficiency-responsive lncRNAs. (Excel file)

Table S4 The GO enhancements of putative lncRNAs' targets in leaves under phosphate deficiency.

GO term	Function	Description	$-\log_{10}(P\text{-value})$
GO:0004499	Molecular function	N,N-dimethylaniline monooxygenase activity	5.03
GO:0050661	Molecular function	NADP binding	3.06
GO:0016746	Molecular function	Transferase activity, transferring acyl groups	2.60
GO:0003840	Molecular function	Gamma-glutamyltransferase activity	2.03
GO:0016758	Molecular function	Transferase activity, transferring hexosyl groups	2.02
GO:0019464	Biological process	Glycine decarboxylation via glycine cleavage system	1.97
GO:0016841	Molecular function	Ammonia-lyase activity	1.85
GO:0006559	Biological process	L-phenylalanine catabolic process	1.55
GO:0004618	Molecular function	Phosphoglycerate kinase activity	1.53
GO:0009607	Biological process	Response to biotic stimulus	1.50
GO:0016161	Molecular function	Beta-amylase activity	1.40
GO:0000272	Biological process	Polysaccharide catabolic process	1.40
GO:0003978	Molecular function	UDP-glucose 4-epimerase activity	1.31
GO:0009058	Biological process	Biosynthetic process	1.31

Table S5 The GO enhancements putative lncRNAs' targets in roots under phosphate deficiency.

GO term	Function	Description	$-\log_{10}$(*P*-value)
GO:0009607	Biological process	Response to biotic stimulus	12.35
GO:0006869	Biological process	Lipid transport	8.03
GO:0005506	Molecular function	Iron ion binding	7.98
GO:0031012	Cellular component	Extracellular matrix	6.86
GO:0004089	Molecular function	Carbonate dehydratase activity	5.60
GO:0004601	Molecular function	Peroxidase activity	5.32
GO:0055114	Biological process	Oxidation-reduction process	5.18
GO:0009055	Molecular function	Electron carrier activity	4.72
GO:0003887	Molecular function	DNA-directed DNA polymerase activity	4.70
GO:0016746	Molecular function	Transferase activity, transferring acyl groups	4.14
GO:0006979	Biological process	Response to oxidative stress	3.79
GO:0030145	Molecular function	Manganese ion binding	3.59
GO:0008152	Biological process	Metabolic process	3.45
GO:0006032	Biological process	Chitin catabolic process	2.97
GO:0016567	Biological process	Protein ubiquitination	2.47
GO:0043039	Biological process	tRNA aminoacylation	2.43
GO:0046470	Biological process	Phosphatidylcholine metabolic process	2.26
GO:0004630	Molecular function	Phospholipase D activity	2.26
GO:0045735	Molecular function	Nutrient reservoir activity	2.24
GO:0006817	Biological process	Phosphate ion transport	2.13
GO:0005315	Molecular function	Inorganic phosphate transmembrane transporter activity	2.13
GO:0009664	Biological process	Plant-type cell wall organization	2.12
GO:0046429	Molecular function	4-hydroxy-3-methylbut-2-en-1-yl diphosphate synthase activity	2.03
GO:0009611	Biological process	Response to wounding	2.01
GO:0006750	Biological process	Glutathione biosynthetic process	2.01
GO:0008168	Molecular function	Methyltransferase activity	1.90
GO:0046274	Biological process	Lignin catabolic process	1.82
GO:0006260	Biological process	DNA replication	1.77
GO:0008081	Molecular function	Phosphoric diester hydrolase activity	1.76
GO:0009058	Biological process	Biosynthetic process	1.70
GO:0005385	Molecular function	zinc ion transmembrane transporter activity	1.60
GO:0005886	Cellular component	Plasma membrane	1.51
GO:0046914	Molecular function	Transition metal ion binding	1.46
GO:0009813	Biological process	Flavonoid biosynthetic process	1.45
GO:0008661	Molecular function	1-deoxy-D-xylulose-5-phosphate synthase activity	1.40
GO:0043044	Biological process	ATP-dependent chromatin remodeling	1.31
GO:0017176	Molecular function	Phosphatidylinositol N-acetylglucosaminyltransferase activity	1.31
GO:0004807	Molecular function	Triose-phosphate isomerase activity	1.31

Fig. S1 The quality score (Q) value of RNA-seq from eight samples.

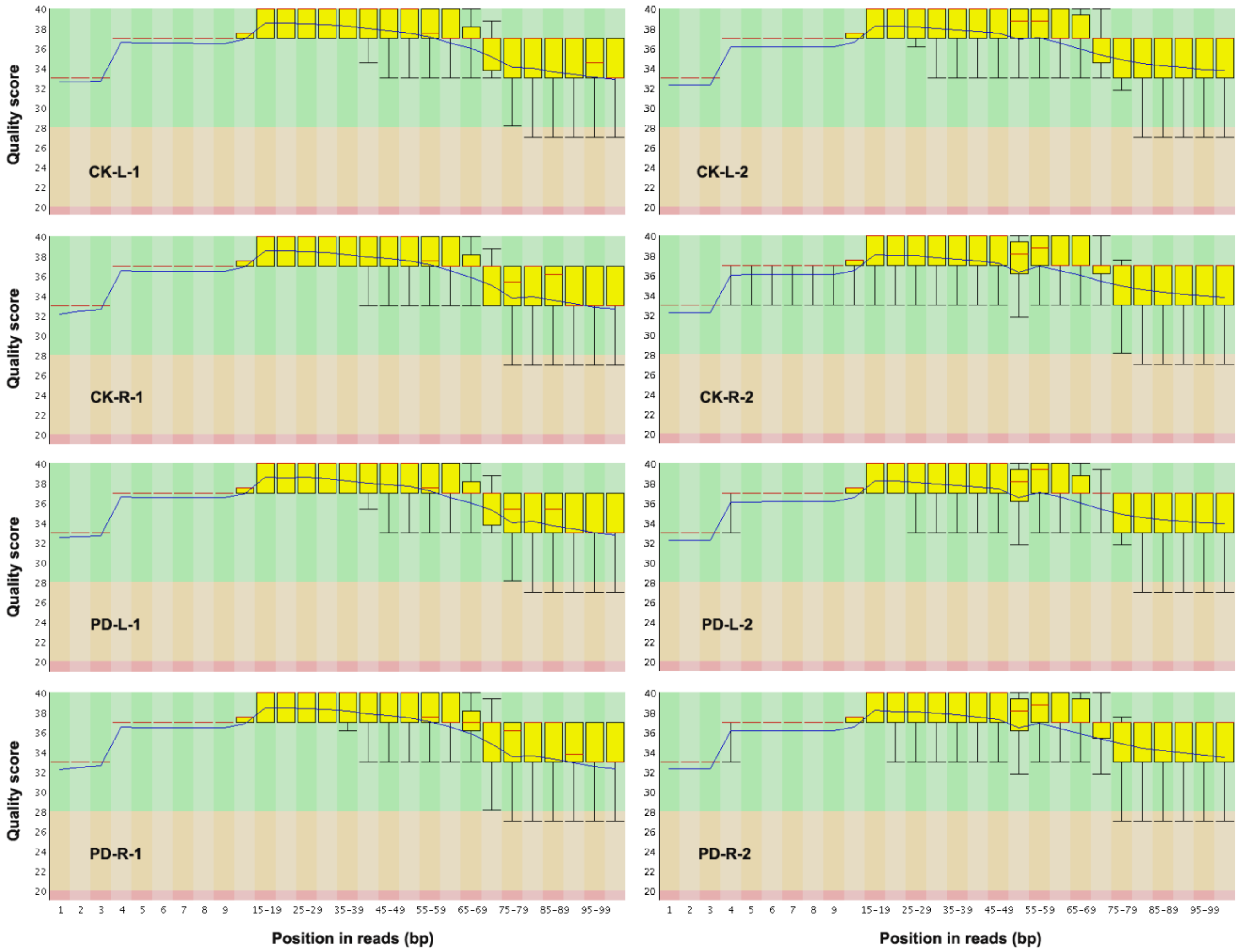

Fig. S2 Alignments of full sequences between *PDIL1* and *Mt4* (A), and between *PDIL2*, *PDIL3* and *Medtr1g074930* (B).

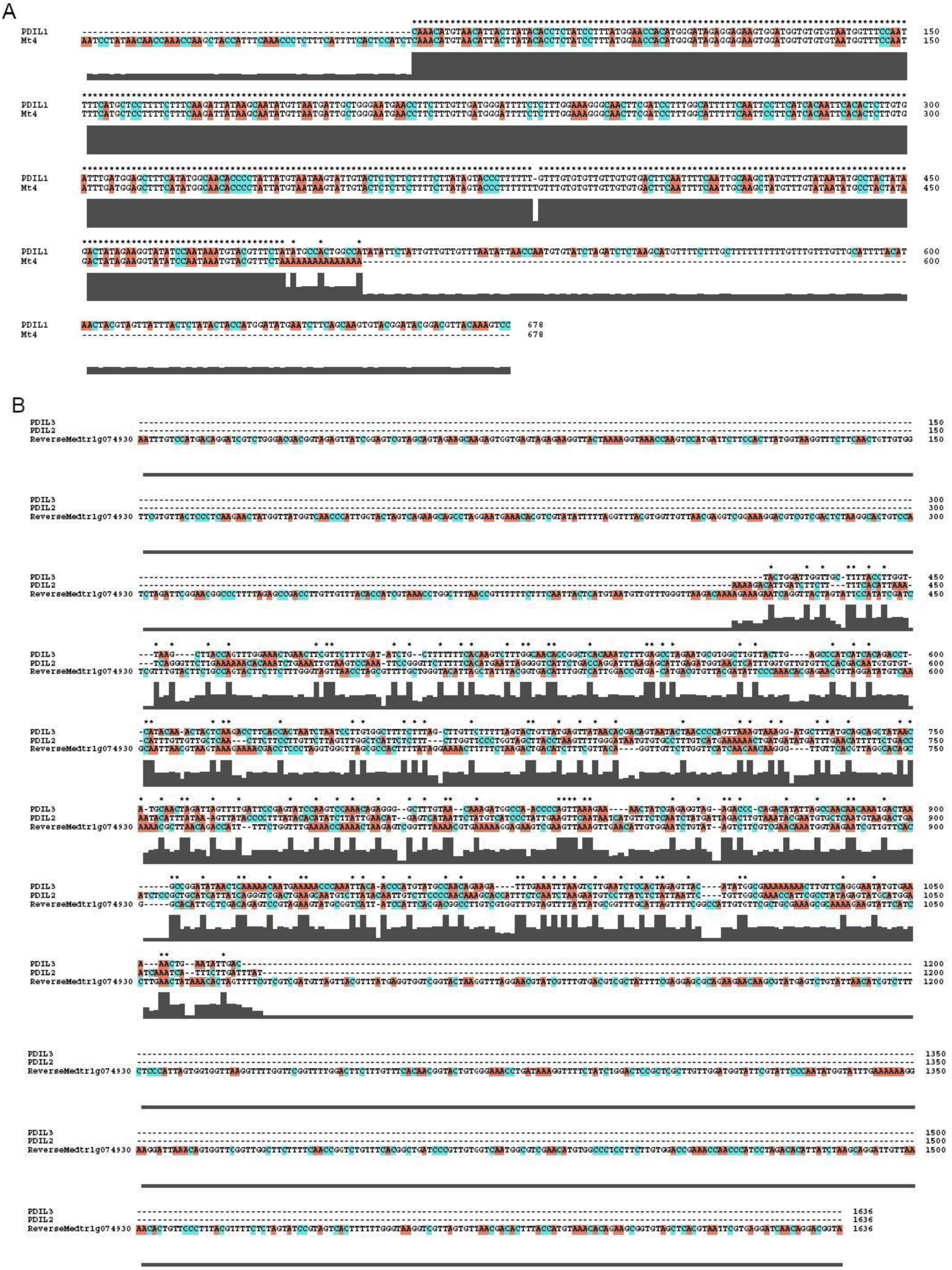

Fig. S3 Alignments of protein sequences between Medtr1g074930 and other phosphate transporters of *Arabidopsis* and rice.

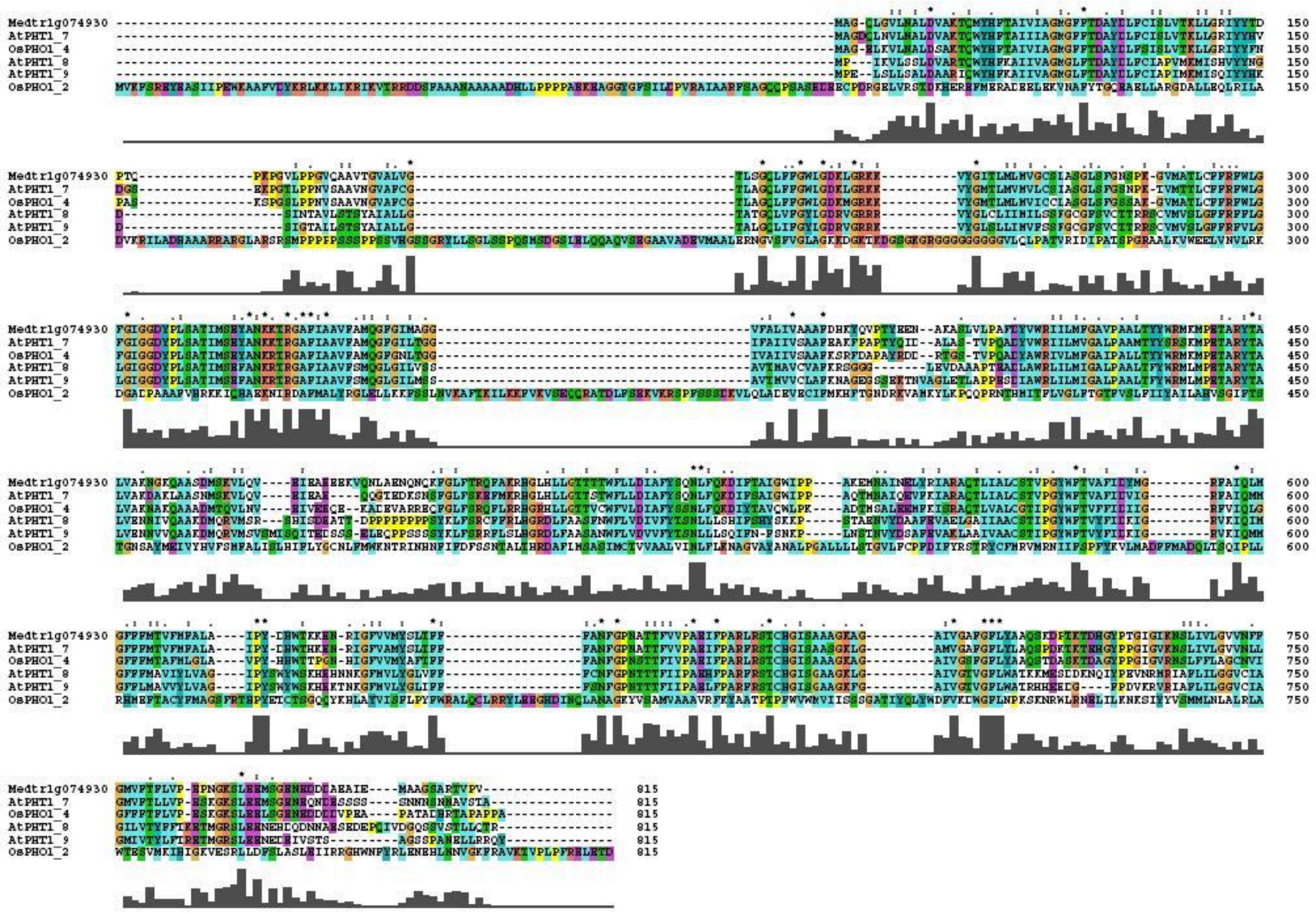

Fig. S4 Identification and confirmation of *pdil2* and *pdil3* mutants. (A) The insertional positions of *Tnt1* in *pdil2* and *pdil3* mutations. The location of primers used for identification of homozygotes was labelled. (B) Identification of homozygotic *pdil2* and *pdil3* mutations. (C) The expression level of *PDIL2* and *PDIL3* in mutants.

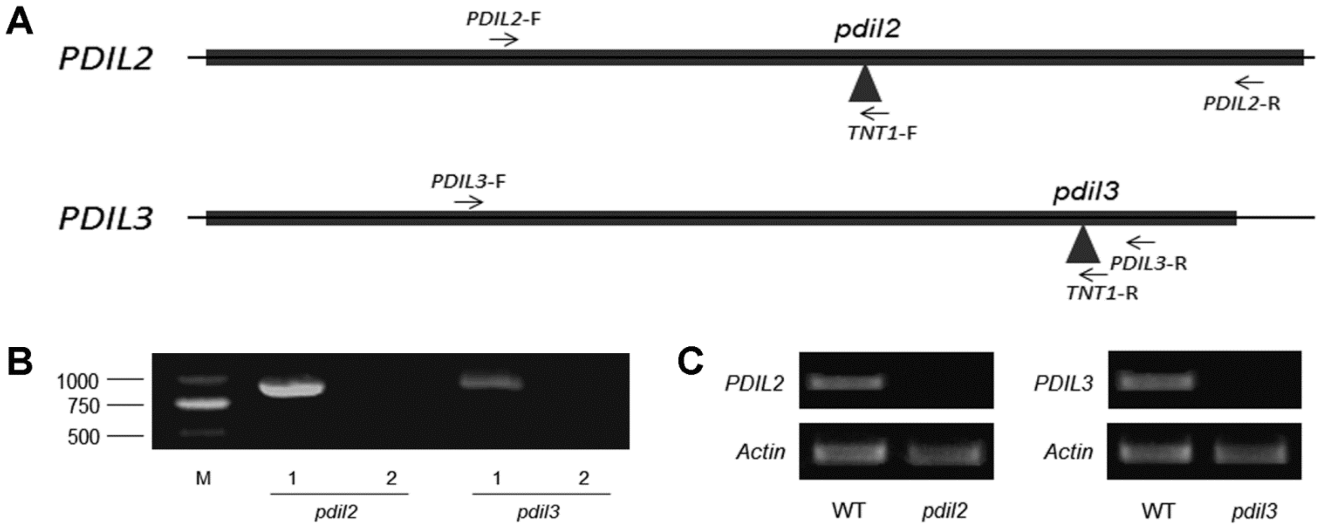

Supplement: supplementary_table_S1_S4_S5_Figures_S1_S4 [file erx384_suppl_supplementary_table_s1_s4_s5_figures_s1_s4.pdf]
